# Exploring College Adjustment in First-Year Gen Z Medical Students and Its Contributing Factors

**DOI:** 10.21315/mjms2022.29.1.12

**Published:** 2022-02-23

**Authors:** Rahma Tsania Zhuhra, Mardiastuti H Wahid, Rita Mustika

**Affiliations:** 1 Master of Medical Education Program, Faculty of Medicine, Universitas Indonesia, Jakarta, Indonesia; 2 Department of Medical Education, Faculty of Medicine, Universitas Indonesia, Jakarta, Indonesia; 3 Department of Clinical Microbiology, Faculty of Medicine, Universitas Indonesia, Jakarta, Indonesia; 4 Medical Education Center, Indonesian Medical Education and Research Institute (IMERI), Universitas Indonesia, Jakarta, Indonesia; 5 Medical Education Collaboration Cluster, Indonesian Medical Education and Research Institute (IMERI), Universitas Indonesia, Jakarta, Indonesia

**Keywords:** ***Keywords:*** medical, students, college adjustment, technology, culture, social adjustment, emotional adjustment

## Abstract

**Background:**

First-year medical students need to adjust to university life to achieve optimal education. Notably, generation Z (Gen Z) students recently admitted to medical school possess unique characteristics that may affect their adjustment. However, limited studies have evaluated the adjustment of Gen Z medical students. In line with this, the present study explores the adjustment process of Gen Z medical students in their first year of study.

**Methods:**

A qualitative phenomenological study was held from January 2020 to October 2020. The respondents comprised first-year students from two medical institutions. Maximum variation sampling was applied to select the respondents. Moreover, 11 focus group discussions (FGDs) with students and 10 in-depth interviews with lecturers were conducted. Curriculum documents were examined, and then the data were analysed thematically.

**Results:**

Three themes were identified: i) domain; ii) process and iii) contributing factors to college adjustment. Academic, social and personal-emotional components of adjustment were included in the domain theme. The process theme consisted of transition, transition-transformation and transformation phases. Meanwhile, the contributing factors consisted of existing and supportive factors. Student characteristics, including demographics, mentality, prior educational experiences and social support, were considered the existing factors, while technology, learning system and well-being constituted the supporting factors.

**Conclusion:**

College adjustment involves various domains, processes and contributing factors that are unique to Gen Z characteristics, technology dependence and culture. Therefore, well-prepared faculties are needed to support the adjustment of Gen Z students.

## Introduction

College adjustment is a psychological process that new students go through in adapting to the new chapter of their early college life. Baker and Siryk as cited in Nyamayaro and Saravanan (1) reported four dimensions of college adjustment, namely, academic, social, personal-emotional, and attachment to the university (1). College adjustment generally takes place when students start their higher education and encounter various obstacles for the first time (2). The first year is more complicated than the following years, as students may face challenges that are associated with being separated from their family, living with new people, and encountering new academic and financial problems that they were not exposed to in high school (3). Crede and Niehorster (2) identified eight key factors contributing to college adjustment: i) demographic characteristics; ii) achievement in the previous school; iii) college life experiences; iv) self-evaluation and personal traits; v) affective state; vi) coping strategy; vii) social support and viii) relationship with parents.

The medical school environment is often a higher-pressure environment compared with other institutions. Medical students are expected to master a vast array of knowledge and skills within a short period. The students’ initial exposure to the medical school environment could negatively impact them physically and mentally. In the United States, 57% of undergraduate medical students experience psychological stress due to maladjustment (4). Maladjustment increases the risk of discontinuation of education, inadequate professional behavior, decreased empathy, poor academic performance, drug abuse and suicidal thoughts (5).

Generation Z (Gen Z, also known as iGen, Gen Tech, Digital natives and Gen Wii) comprises people born between 1996 and 2012 in the United States, with potential minor birth-year differences in other countries (6, 7). Notably, some Gen Z students have recently reached college age and have been admitted to medical schools. They possess unique characteristics compared with their predecessors. They are more connected to technology, particularly social media, though they also value personal experiences. They are creative, entrepreneurial, goal-oriented, realistic, inclined to personalised micro-experiences, and focused on skills and hands-on experiences. They hold high expectations and a sense of justice. Their extensive use of social media leads to a fear of missing out (FOMO). These traits may influence their college adjustment, particularly in adjusting socially, overcoming the transition and persevering in the rigours of higher education (6, 8, 9). These generation-wide characteristics lead Gen Z medical students to be keen on using technology in their education and future patient care. They are interested in using podcasts, websites, simulations, interactive tutorials on YouTube and web-based educational games. They rely more on technology-based patient care and less on face-to-face interactions (10).

Medical schools need to explore these characteristics when preparing for the changes brought about by a new generation of students. Knowledge concerning the college adjustment of first-year medical students is required to support their educational process. Differences in generation-based traits should be used in the decision-making processes involved in optimising student support, attaining high student retention and maximising academic potential (10). This study provides an understanding of the college adjustment of Gen Z medical students during their first year, taking into consideration the contributing factors and experiences related to the adjustment process.

## Methods

We performed a qualitative study with a phenomenological approach at two medical institutions in Indonesia with the same national accreditation level (A) but located on different islands with presumably different cultures. In this study, Gen Z is defined as a generation born between 2001 and 2010 (11). The source population comprised first-year medical students from the two medical institutions with an age range of 17 to 19 years in 2020. We used maximum variation sampling to select the samples; the variations included age, gender, hometown, parents’ occupations and first semester grade point average (GPA). For triangulation, we interviewed medical teachers selected through a maximum variation sampling of gender and teaching experiences; we also studied documents related to the first-year curriculum (12). The total respondents were 59 first-year medical students and 10 medical teachers (12).

We conducted focus group discussions (FGDs) with the medical students and in-depth interviews with the medical teachers until the data were saturated. There were 11 FGDs and 10 in-depth interviews. All the FGDs and in-depth interviews were recorded and transcribed. Thereafter, the verbatim data were analysed thematically using the steps for coding and theorisation (SCAT) model by Otani (13).

## Results

### Characteristics of Respondents

Respondents who participated in this study are described in Table 1.

### Identified Themes

This study identified three primary themes: i) domain of college adjustment; ii) adjustment process and iii) contributing factors. The domain of college adjustment was further classified into academic, social and personal-emotional adjustment. The adjustment process theme was subdivided into the transition, transition-transformation and transformation phases. Two groups of contributing factors were identified, namely, existing factors and supporting factors. These three themes are described below:

#### Domain of College Adjustment

In the academic adjustment portion of this theme, the students reported being challenged by tight schedules, various learning activities, high volume of learning content, development of learning strategies, assessments, language barriers, coordination of multiple tasks and new learning environments. The students shared their experiences related to their academic life in their first year as dramatic events that they needed to overcome.

“I was surprised at first. It was like I was being pushed to learn. I felt overwhelmed with so many subjects; it is not like in high school, where we learned general topics. And I was surprised by the introductory lectures going on.”

In the social adjustment component, the students adapted to the culture, social media use, peer relationships and extracurricular activities on campus.

“I had to adapt to the culture of medical school and the new relationship styles here. I found that they were different from those in high school.”

As young adults, the students also had to undergo a personal-emotional adjustment. They reported experiencing adjustments in attitude, habit, behaviour and even personality.

“I feel more independent because I live far from my parents, so I need to do everything by myself. Now I have to do everything alone, not like in high school when my parents used to wake me up every morning.”

#### Adjustment Process

The adjustment process occurred throughout the entire first year of medical school in different phases. Feelings of separation and alienation were described in the transition phase. The transition-transformation phase occurred halfway through their journey of developing identities as medical students. In the transformation phase, the students accepted and adapted to college life.

“I panicked at the early days, questioning how this is going to be, whether I would be able to do it. But as time goes by, it gets easier, and my friends enjoy college life, too.”

“I was homesick at the beginning of my first year; it was my first experience living far from home. But now, I have adapted well.”

#### Contributing Factors

The existing factors that influence college adjustment were described in five subthemes: i) characteristics; ii) demographic traits; iii) psychological considerations; iv) prior experiences in education and v) social support. In turn, each subtheme had multiple components. Characteristics were identified from established Gen Z traits. They were described as being creative, goal-oriented, self-reliant, realistic, pragmatic, competitive and reflective. They reported having high expectations, supporting justice, focusing on skills, multitasking and valuing personalisation. They were highly connected to social media and at risk for FOMO.

“We search for answers by seeking related journals, for example, in the tutorial session. The Internet provides everything. In a group discussion, we often discuss the learning topics via LINE or WhatsApp, and we search for additional information through Instagram and other social media platforms.”

“I like clinical skill sessions. They strike directly to the point, not just in theory. We understand by learning by doing. For me, it is better to have direct practice sessions rather than just learning theories.”

Some demographic factors that influence adjustment were identified in this study. Those factors included age, religion, language, ethnicity, culture, accommodation and financial situation. The students reported that age played a significant role in college adjustment, as the older students seemed to be more experienced than the younger ones.

“At first, I could not understand the lectures well. I felt like I had less experience than other students who were older than me.”

Notably, the students in Sumatra shared their commentaries on adjusting to a less diverse culture. However, the students in Java did not share the same experience.

“When I am in Pontianak, people are mostly Chinese, unlike here. Religious tolerance is different. Maybe it is kind of less tolerant to ethnicity or tribes. I am surprised; the religious atmosphere is emphasised here (in Sumatra).”

“As a member of a minority group, I do not experience any difficulties related to cultural and religious diversities in my campus environment. I am sure we have well-educated and open-minded people here (in Java).”

Living in a boarding house, being separated from their parents and managing their financial challenges also affected adaptation.

“I worried about being so far from home. I am from Sumbawa, Nusa Tenggara. I had to live in Java as a medical student. It made me nervous and scared.”

“I need to adapt my financial management.”

In addition, psychological considerations, such as self-evaluation, internal and external motivations, affective state, and coping strategies played a role in college adjustment.

“In the first two to three months, I felt like I had impostor syndrome. It was like I did not deserve to be here. Am I going to be stuck here my whole life? But then, my friend showed me that by being a physician, we could help people. When we had our first community assessment session directly with the people in the community, I felt a sensation of satisfaction with helping people. That is the reason I feel this is worth it.”

“Both of my parents are physicians; they want a successor. I am the only child, so I am thrilled to prove that I can become a physician as well.”

“It is easier to learn when I am happy and in a good mood. But when I am tired and upset, I should have some ‘me time’ to recharge my energy. This is my coping mechanism.”

Moreover, prior experiences in education related to high school habits, achievements, and favorite subjects affected college adjustment. In the early part of college, experiences in extracurricular activities, student-teacher relationships and grades were also identified as contributing factors in the students’ adjustment.

“Usually, the people who often took part in competitions in high school have good work ethics and good learning habits. They have high standards because they realise that they have to be the best. They also do not easily give up in their academic achievements.”

“When we come into direct contact with society in a campus event, I have to understand more about social values. I care about people more, and I want to continue my studies in medical school.”

The students also stated that social support from their parents, friends, family and seniors helped them when they struggled during their first year in medical school.

“My parents’ support is one thing that ensures my continuity in medical school. When I get bored, I talk to my parents, asking for advice and opinions.”

“My friends help me adapt well here. We learn and do things together.”

Meanwhile, the supporting factors in students’ adjustment consisted of implementing technology in learning; direct participation, discussion, creative and reflective learning methods, and constructive feedback in the classroom; improvement of facilities, the medical education system, and teaching methods; and students’ well-being. For some students, mental health became an issue. Both teachers and students described the supporting factors in student adjustment as follows:

“One of the teachers showed an embryology video in her lecture session. By watching the video, we were able to imagine the embryology process. The concept was unclear during the first time we learned about the cephalocaudal groove, but with YouTube, we could imagine it. It helps.”

“I think we are supposed to bring blended learning to the education system. I am trying to manage my teaching in online learning now. Teaching with blended learning is the best way for me.”

“One student has a gadget addiction from junior high school, which persists until now. He lags behind and has frequent attendance issues. He is at risk of dropping out in the second or fourth semester.”

“We are insecure inside and have difficulty opening up about our daily struggles in college life. We post our feelings of pride and college activities on social media, but in reality, we are not as good as it seems.”

**Figure 1:**
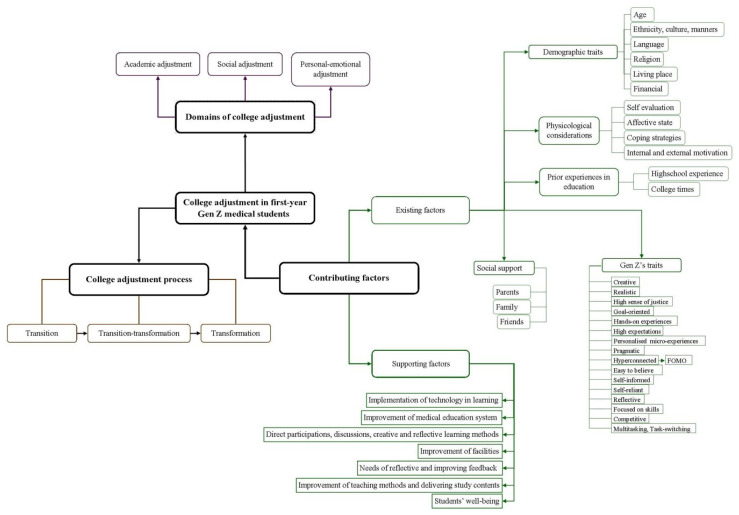
Conceptualisation of the relationship between themes, subthemes and their components in college adjustment

Figure 1 conceptualises the relationship between the themes in student adjustment to the first year of medical school.

## Discussion

We identified three college adjustment domains in this study: i) academic; ii) social and iii) personal-emotional adjustments that students faced in their first year. Students’ attachment to the university was found to be related to social adjustment, which is in contrast to a previous study by Baker and Siryk as cited in Nyamayaro and Saravanan (1), who identified attachment to university as its own domain. Meanwhile, workload and pressure were encompassed by academic adjustment, similar to the findings of Kumar et al. (4).

After examining the respondents’ GPA, most students demonstrated good academic performance, although some had below-average GPA. This may be due to difficulties experienced in adjusting to tight schedules, numerous in-depth subjects and evolving college study habits. Pinyopornpanish et al. (14) described this condition as one of the internal factors influencing academic performance, in addition to low attendance, laziness, games, lack of basic knowledge and poor motivation. Del-Ben et al. (15) concluded that the low academic performance among first-year medical students was caused by a feeling of disconnection from learning topics and activities. The students reported that first-year learning did not meet their expectations for practicing medicine. Our study sheds light on similar feelings amongst students, as described by their predilection to enjoy clinical skill sessions over lectures. Clinical skill sessions contributed to the students’ enjoyment in the acquisition of medical knowledge and their eagerness to continue their studies.

These Gen Z students were described as creative, goal-oriented, self-reliant, realistic, pragmatic, competitive and reflective. These findings are similar to the results obtained by Schwieger and Ladwig (6), Stillman et al. (8), Uhlman (9), Putra (16), and Dwidienawati and Gandasari (17). Their reflective thinking may be related to awareness of failures as chances to improve their performance (18). According to Papadimos (19), reflective thinking among medical students is developed by maintaining daily self-reflection. These traits were also reflected in the personal-emotional adjustment domain through the characterisation of students as being disciplined and independent. They chose active participation and reflective learning methods. They were also self-informed and creatively used technology in learning. However, Gen Z students have been described as having difficulty connecting basic science to clinical applications. According to Talmon (20), Gen Z medical students struggle to connect facts and concepts related to the data they collect. Vogelsang et al. (21) also suggested that adjustment and evolution of ineffective learning styles are imperative for the students to gain the knowledge and experience required for them to become physicians.

The students shared their experiences in using communication technology and internet access in learning, gathering information, creating online discussions and developing creative course sessions. They expressed a desire for the faculty to utilise more technology in the learning process and assessments. Similarly, Eckleberry-Hunt et al. (10) reported that Gen Z medical students wish to use technology in education and patient care. This information could guide faculties in choosing blended learning methods to unite access to information technology and interpersonal collaboration in the real world to fulfill students’ needs (22). The students chose discussion and clinical skill-based sessions because they felt more comfortable in direct participation and real-world scenarios. They preferred video presentations, simulations, case studies and group discussions in learning. According to Bridges in Plochocki (22), Gen Z students are not satisfied with traditional lecture formats; small group learning with mentoring and collaboration sessions help Gen Z students develop critical thinking and professionalism. Stillman in Talmon (20) also stated that Gen Z students place more value on experiences and discussions that are related to practical knowledge.

This study highlights the hyper-connected nature of Gen Z medical students, which is related to their daily use of social media and the presence of FOMO. FOMO detracts from the efficiency of the learning process and task completion. Raacke and Bonds-Raacke (23) found that overuse of social media in information gathering is connected to academic maladjustment. Social adjustment is also disturbed when students exhibit a social relationship with their gadget, not with the real world. Moreover, personal-emotional maladjustment may occur due to stress and physical disturbances introduced by reliance on social media. Jacobsen and Forste (24) stated that gadget use as a multitasking activity increases distractions and disturbs the academic performance of first-year college students. Conversely, Shah et al. (25) showed no clear evidence of the detrimental effects of multitasking in medical students.

Gender was also found to influence academic performance, as reflected by the lower GPAs found in male students. A study by Nawa et al. (26) similarly found that male students tended to have lower GPAs than female students. Notably, Nawa et al. (26) reported that origin, year of graduation and type of entrance exam affected the GPA.

Our study found that age played a role in the adjustment of first-year medical students in terms of their ability to understand the courses and learning experiences. Younger students experienced challenges with academic adjustment associated with a lack of maturity and prior accelerated high school study programmes using different learning styles. Clinciu (27) also reported that students who are 20 years old or older exhibited better college adjustment due to higher levels of maturity.

The students shared that depression and stress affect their learning process. Nyayamaro and Saravanan (1) similarly stated that the stressful academic loads of first-year medical students could disturb adjustment. Coping strategies are needed to maintain success in academic and social adjustment. In this study, most of the students did not easily give up. They used emotion-focused coping, problem-focused coping or both to handle their problems. This finding was in accordance with the study by Perera and DiGiacomo (28), in which they identified the role of coping mechanisms in academic achievement during college adjustment.

Internal and external motivations were identified to be related to students’ adjustment. Internal motivations, such as childhood goals and fondness for particular subjects, made the students happy to be admitted to medical school. Their parents’ will and support dominated external motivation. In this study, parents played a significant role in influencing the students’ decision to enter medical school. Parents who were doctors sought to make their children their successors. Indeed, Chang et al. (29) demonstrated that authoritarian Asian parents play a prominent role in college decisions, particularly because these decisions relate to social status.

The students shared their mental well-being struggles that are related to feelings of unworthiness and their daily struggles in real life and on social media. This might be associated with FOMO, which is a common trend among Gen Z. Hardeman et al. (30) reported the need to increase attention and improve resources to help first-year medical students identify various mental health stressors. It should be emphasised that they are prone to lower mental health states and psychological conditions that could trigger stress, anxiety, self-harm and suicidal thoughts.

To overcome adjustment challenges, the students shared the importance of social support from their parents, families, friends, and seniors. Supportive parents and families are important assets during the adjustment process. Love and Thomas (31) stated that parents have important roles in creating self-esteem and emotional well-being in their children, which boost their academic adjustment in their first year. Gen Z students look up to their families as their financial and emotional support by using communication technologies to stay connected (32). Friends also contribute by working together, sharing, and building a sense of belonging in the early college phase. Hertel (33) reported that students who have friends in college have a better understanding of the campus and feel less depressed than students who do not have friends. Our study also found that involvement in extracurricular and social activities aided in adjustment to university life. Gen Z students also use social media to maintain their social relationships. Similarly, Malay (34) concluded that students primarily use Instagram to maintain their interpersonal relationships during social adjustment. However, they still need face-to-face interactions to maintain their personal development and soft skills. Stillman et al. (8) stated that Gen Z students tend to participate in well-guided face-to-face interactions.

Moreover, improvement of facilities was necessary in the students’ opinion. This was related to the sense of well-being that influences academic adjustment. Lack of facilities may disturb the learning process and cause discomfort. According to Stillman et al. (8) and Eckleberry-Hunt et al. (10), well-being is one of the critical considerations of Gen Z students. Students are not hesitant to express their opinions regarding the education system and teaching performance. Thus, Miller and Mills (35) proposed an effective teaching method for Gen Z students that involves prioritising the expression of caring for student success.

Enrolling in medical school involves rigorous curriculum, pressure, and achievement in medical ethics, skills, attitudes and knowledge to ensure the best care for patients. In this regard, the culture of medical school is different from that of other institutions due to the high stakes and demanding academics. Indonesia has 83 medical schools located throughout its 34 provinces (36), and there are differences in culture and ethics in medical education across these institutions. According to Pratt in Shamim et al. (37), in Asia, the differences are influenced by religious and local cultures. Thus, Western standards for medical education cannot be applied for these reasons.

Our findings indicate that social adjustment was influenced by ethnicity, culture and local manners. Students from Sumatra were required to adjust to the local culture that relies on tradition and the presence of strong religious values in daily life, including through communication (such as everyday language use), social relationships, manners, clothes and so on. Anwar et al. (38) stated that educational institutions, including universities, maintain local wisdom and cultural resistance as part of the institutional culture. The students from outside Sumatra, such as those from metropolitan areas and diverse cities in Java and other islands, were surprised and overwhelmed by their initial exposure to college life. However, the students attending school in Java did not share the same experience in relation to local culture. In the metropolitan environment where this study’s institutions are located, the population is heterogeneous and diverse. Differences in culture and religion are not necessarily notable. According to Bowman et al. (39), different ethnic and demographic backgrounds could hinder the process of college adjustment, although a sense of belonging to the college environment can overcome it. A sense of belonging gained through college experiences, involvement in extracurricular activities, and social support can facilitate adjustment.

There is a variation of the adjustment process found in this study compared with the findings of Van Gennep in Nubia-Feliciano (40). Van Gennep divided the college adjustment process into transition and transformation phases, with the transition process consisting of its own three components: i) separation;, ii) transition and iii) incorporation. This study simplified the process into transition, transition-transformation and transformation. The transition process is described by separation from parents, friends, and a familiar environment and difficulties in learning due to sadness, depression and anxiety. Meanwhile, the transition-transformation process occurs throughout the students’ journey amid their efforts to accept their situation and adjust to college life. Finally, the transformation process is described as students enjoying medical school, being happy with their education, and feeling safe. According to Bowman et al. (39), the college adjustment process moves in the proper direction over time. However, it is not a linear process, with progress and challenges occurring concurrently in relation to college adjustment factors and supporting factors.

## Limitations

This was a qualitative study with a phenomenological approach performed specifically in two medical institutions in Indonesia. Hence, institutional and local cultures, learning activities and normal practices may be different in other regions. Further studies are needed to gain a global perspective of Gen Z medical students and their adaptation.

### Conclusion

The influence of Gen Z attributes on the college adjustment process of first-year medical students necessitates the application of novel approaches other than those adopted in previous generations, even if the rigours of medical school remain the same. Local and institutional cultures play significant roles in college adjustment. College adjustment is a two-way effort that requires contributions from both the students and the faculty. Knowledge concerning the adjustment process is necessary to overcome the challenges during the critical period for first-year medical students. Faculties should consider curriculum evaluation, staff development and facilities improvement to meet the needs of Gen Z medical students. Conducting regular follow-ups through well-developed student support systems is also essential for the students’ well-being.

## Figures and Tables

**Table 1:** Characteristics of respondents

Respondents	Characteristic	Description	Number
Students	University location	Sumatra	35
		Java	24
	Age (years old) in 2020	17	3
		18	13
		19	43
	Gender	Male	29
		Female	30
	Hometown island	Sumatra	26
		Java	28
		Kalimantan	2
		Sulawesi	2
		Nusa Tenggara	1
	Living with	Parents or family	15
		Boarding house	44
	Parents’ occupation	Physician	11
		Non-physician	48
	First semester GPA	Below average	17 (12 males)
		Within or above average	42

Teachers	University location	Sumatra	6
		Java	4
	Gender	Male	4
		Female	6
	Teaching experience	Junior (less than 10 years)	3
		Senior (more than 10 years)	7
